# Mathematics Achievement in Women With and Without ADHD: Childhood Predictors and Developmental Trajectories Into Adulthood

**DOI:** 10.1177/00222194241301044

**Published:** 2025-01-06

**Authors:** Laura N. Henry, Rachel A. Gross, Stephen P. Hinshaw

**Affiliations:** 1 University of California-Berkeley, USA; 2 Indiana University-Bloomington, USA; 3 University of California-San Francisco, USA

**Keywords:** attention-deficit/hyperactivity disorder (ADHD), mathematics, longitudinal

## Abstract

Youth with attention-deficit/hyperactivity disorder (ADHD) often exhibit impairments in mathematics, but long-term math development into adulthood, particularly in females, is underexplored. We characterized trajectories of math achievement in girls with ADHD and an age- and ethnicity-matched comparison sample from childhood through early adulthood across four waves and examined childhood cognitive predictors (global executive functioning, working memory, processing speed) of trajectories. The ethnically and socioeconomically diverse sample consisted of 140 girls with carefully diagnosed ADHD and 88 neurotypicals, ages 6 to 12 years at baseline, from the United States. Using latent growth curve models, we examined predictors of 16-year math achievement trajectories. In both the ADHD and neurotypical groups, scores declined over time; rates of change did not differ significantly. Yet in the ADHD sample, math difficulties (defined as scores at least 1 *SD* below the national average) increased notably over time, with many such difficulties emerging after childhood. By adulthood, nearly half of women with ADHD exhibited clear math difficulties. Worse baseline global executive functioning predicted slower math growth over time. Girls with ADHD may benefit from math supports *before* concerns emerge or worsen after childhood. Additional research on preventive interventions for math difficulties, including investigation of executive functioning, is warranted.

Characterized by persistent inattention and/or hyperactivity-impulsivity that interfere with development and functioning, attention-deficit/hyperactivity disorder (ADHD) is highly prevalent in the United States (Centers for Disease Control and Prevention [CDC], 2019). The first reported concerns of ADHD are often school-related (Loe & Feldman, 2007), so academic functioning is a key impairment for individuals with ADHD. For children diagnosed with ADHD, strong cross-sectional and longitudinal evidence reveals impaired academic outcomes (e.g., Di Lonardo Burr et al., 2022; Hinshaw, 2002; Lawrence et al., 2021).

Less is known about whether children with ADHD show persisting academic difficulties over time, and how these difficulties might fluctuate across development. Longitudinal studies to date reveal increasing academic difficulties across adolescence (Di Lonardo Burr et al., 2022; Lawrence et al., 2021) and heterogeneous academic trajectories (DuPaul et al., 2017). Several questions remain unaddressed: How might academic skills potentially develop from childhood *to adulthood*? From childhood, can we predict future lags in academic skills to inform prevention efforts? Might gender play a role in long-term academic development for children with ADHD?

Such understanding is important given the utility of adult academic skills for higher-education pursuits and daily life functioning (e.g., math skills in adults have been linked to financial decision-making/management, wealth, filing income taxes, reading graphs/charts, following recipes, and using measuring devices; Agarwal & Mazumder, 2013; Johnson & Blalock, 1987; Siegel, 1974). Moreover, given the loss of external supports for individuals with ADHD during the transition from childhood to adulthood (e.g., Treuer et al., 2016), academic trajectories may be informative to families hoping to plan for future outcomes. Childhood prediction of later trajectories is also important to investigate given the importance of earlier rather than later intervention for learning challenges (Fletcher et al., 2018).

## Math Achievement in Girls and Women With ADHD

Herein, we examine math achievement trajectories from childhood to adulthood and their childhood predictors in an all-female sample. We focus on females for three important reasons. First, research on girls and women with neurodevelopmental disorders such as ADHD lags behind research on males (Hinshaw et al., 2022). Second, women with ADHD face barriers to academic development not only from neurodevelopmental disability but also from gender. Although girls perform well in math in school when equal opportunities are available (e.g., Else-Quest et al., 2010), stereotypes, societal perceptions, and lack of societal gender parity in mentorship/careers/school opportunities may impede long-term math performance and pursuit of continued higher math education (e.g., Else-Quest et al., 2010; Good et al., 2008). Thus, girls with ADHD deserve extra focus on their long-term math development. Third, prior research suggests that academic achievement plays an important role in their long-term well-being. Adolescent academic performance is a mediator in the pathway between childhood cognitive impairment and comorbid adult psychopathology in girls with ADHD (Owens & Hinshaw, 2016) and in the pathway between childhood ADHD and young-adult unplanned pregnancy (Owens & Hinshaw, 2020). Academic achievement is also a partial mediator of the relation between childhood ADHD symptoms and young-adult intimate partner victimization (Guendelman et al., 2016). Strengthening academic achievement could help protect against such outcomes.

Consistent with findings in males, in childhood, girls with ADHD score lower on standardized achievement tests than neurotypical girls (Hinshaw, 2002). We focus longitudinally on *mathematics* specifically because, in addition to the barriers to long-term math development faced by women (described above), initial evidence suggests a unique pattern of longitudinal impairment in this domain for girls with ADHD: Namely, a *decline* in math achievement standard scores from childhood to adolescence among girls with ADHD despite improvement in math in a matched comparison group and despite no group differences in reading progression over time (Hinshaw et al., 2006). Yet it remains unclear how math skills continue to develop in girls with ADHD and whether this lag in math performance continues through adulthood.

## Cognitive Predictors of Math Developmental Trajectories

Given the potential benefits of early intervention for learning challenges, research on potentially malleable child factors that predict girls’ later math trajectories is a priority. Research on math and ADHD suggests that cognitive functions are likely candidates. Indeed, in the literature on ADHD, executive functioning (EF) has received widespread attention, given that many individuals with ADHD display clear deficits in this area (e.g., Willcutt et al., 2005). Although the nature of EF is still debated, we define EF as a multidimensional construct composed of diverse yet interrelated cognitive control mechanisms that support goal-directed behaviors (e.g., planning, response inhibition, sustaining attention, working memory [WM]; e.g., Diamond, 2013; Friedman & Robbins, 2022; Pennington & Ozonoff, 1996). Based on prior evidence, we highlight one subcomponent of EF (WM, the ability to hold information in mind long enough to complete operations), plus a measure of global EF (involved in higher-order goal-oriented behaviors, such as planning and strategizing).

Linked with prefrontal brain regions and their intricate interconnections elsewhere (e.g., Friedman & Robbins, 2022; Pennington & Ozonoff, 1996), EFs play different roles in the learning of math facts, concepts, and procedures over time (Cragg & Gilmore, 2014). Previous longitudinal research on links between childhood EFs and adolescent outcomes in girls with ADHD reveals that, out of several childhood EFs (global EF, WM, response inhibition, sustained attention), only WM and global EF predicted late-adolescent math performance (Miller et al., 2012). Yet it is unknown whether these childhood EFs predict math skills into adulthood. In fact, as math complexity increases across adolescence and adulthood, these EFs may be increasingly needed to perform tasks such as planning steps to approach math problems and to hold complex information in mind while performing steps.

Finally, processing speed (PS) is another cognitive capacity linked with both ADHD (e.g., Condo et al., 2022; Cook et al., 2018) and academic achievement (e.g., Caemmerer et al., 2018). Defined as the capacity to perform mental operations quickly (e.g., Bull & Johnston, 1997; Cook et al., 2018), PS is associated with white-matter tracts in temporal, parietal, and frontal lobes (Turken et al., 2008). It supports math calculation in typical development (e.g., Bull & Johnston, 1997; Caemmerer et al., 2018), even on untimed tasks (Child et al., 2019; Willcutt et al., 2013). Both PS deficits and academic difficulties are linked specifically with the inattentive symptom domain (e.g., Condo et al., 2022). Still, long-term prospective relations between PS and math achievement in ADHD are unknown. As academic demands increase over time, math learning processes might be increasingly affected by slow PS, such as note taking, keeping up with classroom lectures, and completing assignments in a timely manner. Thus, childhood PS difficulties might also predict increasing math difficulties over time.

## Present Study

The goals of the present study are as follows: (a) Characterize the development of math achievement in girls with childhood diagnosed ADHD versus neurotypical comparison girls from childhood to early adulthood. Because of higher rates of academic difficulties, we predict that girls with ADHD will show slower growth over time in math performance compared to girls with typical development. (b) Identify childhood cognitive predictors of math trajectories, including WM, global EF, and PS. Given the evidence reviewed above, we predict that difficulties in all three domains will predict slower growth in math performance over time.

## Method

### Participants and Procedure

The Berkeley Girls with ADHD Longitudinal Study began in the late 1990s to redress a lack of research on girls and women with ADHD (Hinshaw, 2002). A sample of 140 girls with ADHD and an age and race/ethnicity matched comparison sample (88 girls) enrolled in the study via research-oriented summer camps (age range = 6–12). Recruited from a variety of medical/mental-health/community sources, girls lived in homes in which English was the primary language and did not have intellectual disabilities, psychotic symptoms, clear neurological damage/injury, or medical issues precluding summer camp involvement (see below under Measures for inclusion criteria for the ADHD and comparison groups). Selected to reflect regional demographics, the sample included 53% White, 27% Black, 11% Latina, and 9% Asian-American girls. Socio-economic status (SES) was highly variable, ranging from receipt of public assistance to professional status. For further detail on participant demographic and background characteristics, see the online supplemental materials and Table S1 as well as Hinshaw (2002).

The summer programs involved extensive, multisource and multi-informant data collection, including measures of parenting and socioenvironmental constructs, objective tests of neurocognitive ability, and observational data. For girls receiving stimulant medications, testing was conducted during unmedicated periods. Prospective follow-up data collection occurred every 5 to 6 years. At Wave 2, ages ranged from 11 to 18 years; at Wave 3, 18 to 23 years; and at Wave 4, 23 to 29 years. Retention was strong (92%–95%) at each wave. Girls participating in follow-up were similar to those who did not (e.g., no significant racial/ethnic differences, differences in mother’s report of child ADHD symptoms, or differences in child academic achievement), but the retained sample at Wave 4 was higher in SES, lower in teacher reported ADHD symptoms, and higher full-scale intelligence quotient (IQ) than those who were lost to follow-up (see Owens & Hinshaw, 2016).

### Measures

Measures were from Wave 1, except math scores, which were collected at all four waves. See the supplemental materials/Table S1 for means and standard deviations of baseline study variables in each group.

#### ADHD Diagnosis

Initial study screening included parent and teacher ratings on the Swanson, Nolan, and Pelham Rating Scale, 4th edition (SNAP-IV, Swanson, 1992). Afterwards, trained clinicians confirmed ADHD symptoms using the Diagnostic Interview Schedule for Children, 4th edition (DISC-IV) parent interview, which has test–retest reliability for ADHD diagnosis (measured in a clinical sample) of kappa = 0.79, as well as sufficient evidence of the validity of previous DISC versions from which the DISC-IV was adapted, including an inter-interviewer agreement (between lay interviewers and clinician interviewers in a community sample) of kappa = 0.60 (see Schwab-Stone et al., 1996 and Shaffer et al., 2000, for additional detail). Final study eligibility for the ADHD sample involved meeting ADHD criteria on both parent/teacher-reported ratings and the DISC-IV. The comparison group did not meet criteria for ADHD (see Hinshaw, 2002).

#### SES

We scaled maternal education from 1 to 6 (1 = *less than 8th grade*, 2 = *some high school*, 3 = *high school or GED completed*, 4 = *some college*, 5 = *BA/BS completed*, 6 = *advanced or professional degree*) (*M* = 4.79, *SD* = 0.95) and family income from 1 to 9 (1 = *less than $10,000*, 2 = *$10–20,000*, 3 = *$20–30,000*, 4 = *$30–40,000*, 5 = *$40–50,000*, 6 = *$50–60,000*, 7 = *$60–70,000*, 8 = *$70–75,000*, 9 = *more than $75,000*) (*M* = 6.43, *SD* = 2.57). As in prior research from this study (e.g., Owens & Hinshaw, 2016), SES comprised the standardized average of maternal education and income.

#### IQ

Administered by highly trained graduate students, the well-validated Wechsler Intelligence Scale for Children, Third Edition (WISC-III, Wechsler, 1991) measured IQ. Full-scale IQ reliability coefficients range from .94 to .97 across different age groups.

#### PS

From the well-validated WISC-III, we used the PS Factor, which consists of the Symbol Search subtest (visually detecting target symbols from a set as quickly and accurately as possible), which has a test-retest reliability of 0.76, and the Coding subtest (using a key to write a series of symbols corresponding to a series of boxes quickly and accurately), which has a test–retest reliability of 0.79 (note that split-half internal consistency metrics are not appropriate for speeded tests such as those that comprise the PS Factor, so the WISC-III manual instead reports test–retest reliability for these subtests, Wechsler, 1991). These tasks leverage visual processing, thinking, and motor speed during relatively simple nonverbal problems.

#### WM

We used the WISC-III Backward Digit Span score from the Digit Span Subtest (which has a split-half subtest reliability of 0.85, Wechsler, 1991), which involves listening to series of numbers and repeating them backwards. It requires leveraging WM to hold the information in mind and manipulate the information for the intended result.

#### Global EF

The Rey–Osterrieth Complex Figure Test (Rey, 1941) involves copying a complex figure. The copy trial—in which participants draw the figure on a blank page while seeing the figure—was used in the present analyses; it captures several skills such as visual-motor integration, organization/planning, WM, and cognitive control, so we consider it a measure of global EF. Multiple studies provide evidence that this task indexes EF. Early examinations distinguished patients with frontal lobe lesions from those without (Lezak, 1995), and scores are correlated with other measures of EF (e.g., Sami et al., 2004; Watanabe et al., 2005). Several scoring procedures exist; measurement varies vastly by scoring procedure, as some scoring procedures possibly detect visuo-spatial skills instead of EF (e.g., Weber et al., 2013). We used the error proportion scoring system (EPS: total errors/total number of moves), which taps global EF, highlighting planning skills (see Sami et al., 2004 for details and validity). The EPS is an adapted version of the developmental scoring system error score (Bernstein & Waber, 1996) enhanced to (a) avoid the floor effects found with the developmental scoring system, (b) capture more detail from disorganized drawings, and (c) highlight planning skills by creating a *proportion score* (rather than just reporting the number of errors). The EPS scores are significantly correlated with other EF measures, most strongly with a measure of nonverbal planning skills, and are more sensitive to EF impairment in children with ADHD than the developmental scoring system (Sami et al., 2004). To avoid confounding, we include visuo-spatial abilities (measured by the WISC Performance IQ Scale; PIQ) as an additional covariate in analyses including this measure. Higher EPS scores indicate more errors—i.e., worse global EF skills. Intraclass correlations of EPS between pairs of three primary scorers ranged from 0.87 to 0.97.

#### Academic Achievement

From the validated Wechsler Individual Achievement Test, either first or second edition (WIAT; Wechsler, 1992, 2001), the Math Reasoning subtest measures the skill of math problem-solving. Compared to basic math operations, math problem-solving recruits a wider variety of cognitive capacities (e.g., Swanson, 2011) and features daily applications of math (e.g., money or telling time). This subtest does not impose a strict time limit. The Word Reading subtest (used as a covariate in Aim 2 supplementary models) measures word recognition, decoding, and phonological awareness.

At Waves 1 and 2, we used the first edition of the WIAT. Wave 3 included a mix of the first edition and second edition (WIAT-II), because the second edition was released during this time period; Wave 4 included only the WIAT-II. The correlation between WIAT I and II Math Reasoning subtests is 0.82 (Wechsler, 2001); the WIAT I mean standard score (108.87) is 3.85 points above the WIAT II mean standard score (105.02). After consultation with a psychometrician working with the test developer, we adjusted all WIAT scores (first edition) by subtracting 3.85 from each WIAT mean score so that the two versions could be comparable. Test–retest reliability for the WIAT (first edition) Math Reasoning, averaged across grades, was 0.89 (Wechsler, 1992). The test–retest reliability of the Math Reasoning subtest of WIAT-II, averaged across the different validity-sample age groups, was 0.94 (Wechsler, 2001).

In trajectory analyses, raw scores are typically optimal to illustrate growth over time in a domain. Yet because raw scores were not comparable between test editions, we instead used standard scores (i.e., status relative to population-based sample). Although doing so limits answering certain questions about skill growth, standard scores enabled us to answer questions about change over time in the status of math skills relative to national norms.

#### Education Level

Included in some secondary analyses (see below), participant education level at Wave 4 was coded on a six-point scale (range = 1–6): not completing high school; high-school diploma; associate’s degree (Community College, for example); trade certificate program (beautician, building trades, etc.); BA or BS, secondary degree at MA or MS level; or completed secondary degree at PhD, MD, law degree, or similar (*M* = 2.43, *SD* = 1.46, Min = 0, Max = 5).

### Data-Analytic Plan

Longitudinal achievement data were collected across the four waves, with each wave encompassing a 5- to 7-year age span (e.g., the Wave 2 age range is 11–18 years old). Trajectory analyses with each wave representing a single point in time would lack specificity (e.g., Wave 2 could represent preadolescence or late adolescence), particularly given the substantial changes in cognitive functioning that occur across adolescence (e.g., Gordon & Hinshaw, 2020). Following the recommendations of Bollen and Curran (2006) for developmental restructuring of longitudinal data for structural equation modeling (SEM), and consistent with prior research in our sample (Porter et al., 2021), we restructured the data set by developmental periods (childhood: *M*_age_ = 8.7, range = 6.6–10.6; early adolescence: *M*_age_ = 12.2, range = 10.6–14.0; late adolescence: *M*_age_ = 16.5, range = 13.8–19.3; emerging adulthood *M*_age_ = 21.4, range = 19.0–25.0; mid/late 20s: *M*_age_ = 26.3, range = 24.0–30.0) to reflect developmental period rather than wave of assessment. Missing data were created by this process, so we used these developmental age clusters only in SEM analyses, as SEM handles missing data via full information maximum likelihood FIML estimation (see below).

#### Aim 1: Does Childhood ADHD Status Predict Growth in Math Achievement?

We used latent growth curve models (LGCMs) to characterize growth in math achievement. Analyses were conducted in Mplus version 8.2 (Muthén & Muthén, 2017). First, we conducted an unconditional growth curve model to determine what shape of growth best fits the data without the inclusion of predictors. Next, childhood ADHD diagnostic status was included as a predictor of the latent intercept and latent slope, along with covariates (SES, full-scale IQ, see below under “Covariates” for rationale), to examine whether the rate of change in math scores of girls with ADHD significantly differed from that of the neurotypical comparisons.

Missing data were handed via FIML estimation. The FIML allows participants to remain in analyses as long as they provide data on math achievement on at least one measurement occasion; it has been shown to provide unbiased parameter estimates (Bollen & Curran, 2006; Enders & Bandalos, 2001). It is highly compatible with developmental period-based data restructuring (Bollen & Curran, 2006).

To contextualize findings on change in math over time clinically, we also report the proportion of women in each group with math difficulties at each wave, the proportion of girls without childhood math difficulties who developed math difficulties by adulthood, and the proportion of girls with childhood math difficulties whose math difficulties remitted by adulthood. There seems to be agreement among experts that achievement scores above the 25th percentile, which is classified by the WIAT as the “Average” range, would not be clinically meaningful difficulties (Fletcher et al., 2018). Still, there is disagreement among experts about what constitutes a meaningful learning difficulty below the “Average” range. Thus, we calculated math difficulties via several criteria. Evidence suggests that achievement test score cutoffs below the 16th percentile (standard score = 85) best identify learning difficulty-associated functional impairment (Brueggemann et al., 2008), so our criteria included math scores (a) at least 1 *SD* below the national mean (standard score <85), (b) at least 1.5 *SD* below the national mean (standard score <78), and (c) at least 2 *SD* below the national mean (standard score <70). This “low achievement” model of identifying learning difficulties offers stronger predictive ability/reliability/validity/sensitivity than other models (i.e., IQ-achievement discrepancy) (e.g., Brueggemann et al., 2008).

#### Aim 2: Are Childhood Cognitive Capacities Related to Growth in Math Achievement?

##### Preliminary Regression Models

We first investigated whether each predictor was related to long-term math performance by conducting initial regressions between childhood (Wave 1) predictors and adult (Wave 4) math scores. To reduce risk of Type 1 error, we applied the Benjamini–Hochberg alpha corrections (Benjamini & Hochberg, 1995) with a false discovery rate of 0.05. Given significant regressions, we included predictors in the LGCM models.

We then addressed two secondary exploratory questions for significant predictors of Wave 4 math. (1) Are these predictive relations similar in girls with and without ADHD? Here, we reconducted regression analyses with childhood diagnosis as a potential moderator. (2) Are these predictors related to math specifically or academic skills more broadly? Here, we reconstructed regression analyses with Wave 1 WIAT reading as an additional covariate. The same alpha corrections and covariates were applied as in our primary analyses.

##### LGCM

Given significant relations with math in regression models, we used separate LGCMs to examine predictors of math growth parameters. Missing data were handled via FIML.

##### Covariates

For all analyses in Aim 1 (Does ADHD predict math growth?) and Aim 2 (Do cognitive skills predict math growth?), SES was included as a covariate given well-documented evidence of its association with academic scores (e.g., Sirin, 2005). For all models in Aim 2 only, diagnostic group could be a confound (e.g., those with more EF deficits are more likely to have ADHD). Thus, we included ADHD status as an additional covariate, consistent with several previous studies of cognitive ability from our sample (e.g., Miller et al., 2012).

For Aim 1, it is debatable whether IQ should be included in the model given the possible risk of overcontrol (see Miller & Chapman, 2001). However, including it as a covariate helps tease apart the role of ADHD from low IQ in math growth, given that both are associated with academic achievement (e.g., Calub et al., 2019). Deliberating between options, we include IQ as a covariate in our primary analysis to be stringent, and also report supplemental analyses reconstructing the model without covarying IQ (see supplemental materials).

For Aim 2, core debate exists about whether IQ should be included as a covariate in studies of cognitive skills in neurodevelopmental disorders. Indeed, there is a clear risk of statistical “overcontrol,” given substantial shared variance between IQ and different cognitive skills (Dennis et al., 2009). Yet because various cognitive abilities and IQ are interrelated, low intelligence may confound findings on cognitive skill predictors. Weighing the options, we covaried childhood ADHD status (see above for rationale) but not IQ (given overlap between ADHD and IQ, see Miller & Chapman, 2001) in primary analyses. Yet to be as stringent as possible, we additionally include PIQ as a covariate in models with global EF, given the risk of confounding visuo-spatial skills with the global EF measure. We also report supplementary LGCM analyses covarying IQ (but not ADHD status; see Table S2 and supplemental materials). In sum, we report analyses both with and without covarying IQ for both aims.

Another potential covariate that requires careful consideration—because of potential overcontrol—is participant education level (i.e., do those with better cognitive abilities or those without ADHD pursue more higher education, by which their math skills increase over time?). However, this poses a chicken-and-egg question: Do trajectories of math predict higher-education pursuits, or vice versa? To prevent overcontrol, we do not include education level in primary analyses. When significant predictors of slope emerge in primary analyses, we reconstruct them, adding education level as an additional covariate to explore its potential role.

Finally, we examined correlations between global EF, WM, and PS. In the case that our regressions (each predictor considered in a separate model) reveal more than one significant cognitive predictor of math outcome, *and* if those predictors are correlated, we conducted secondary analyses to explore independent versus shared contributions of each cognitive predictor by examining correlated measures in the same model.

## Results

### Aim 1

#### Math Standard Scores Over Time

**Figure 1.**
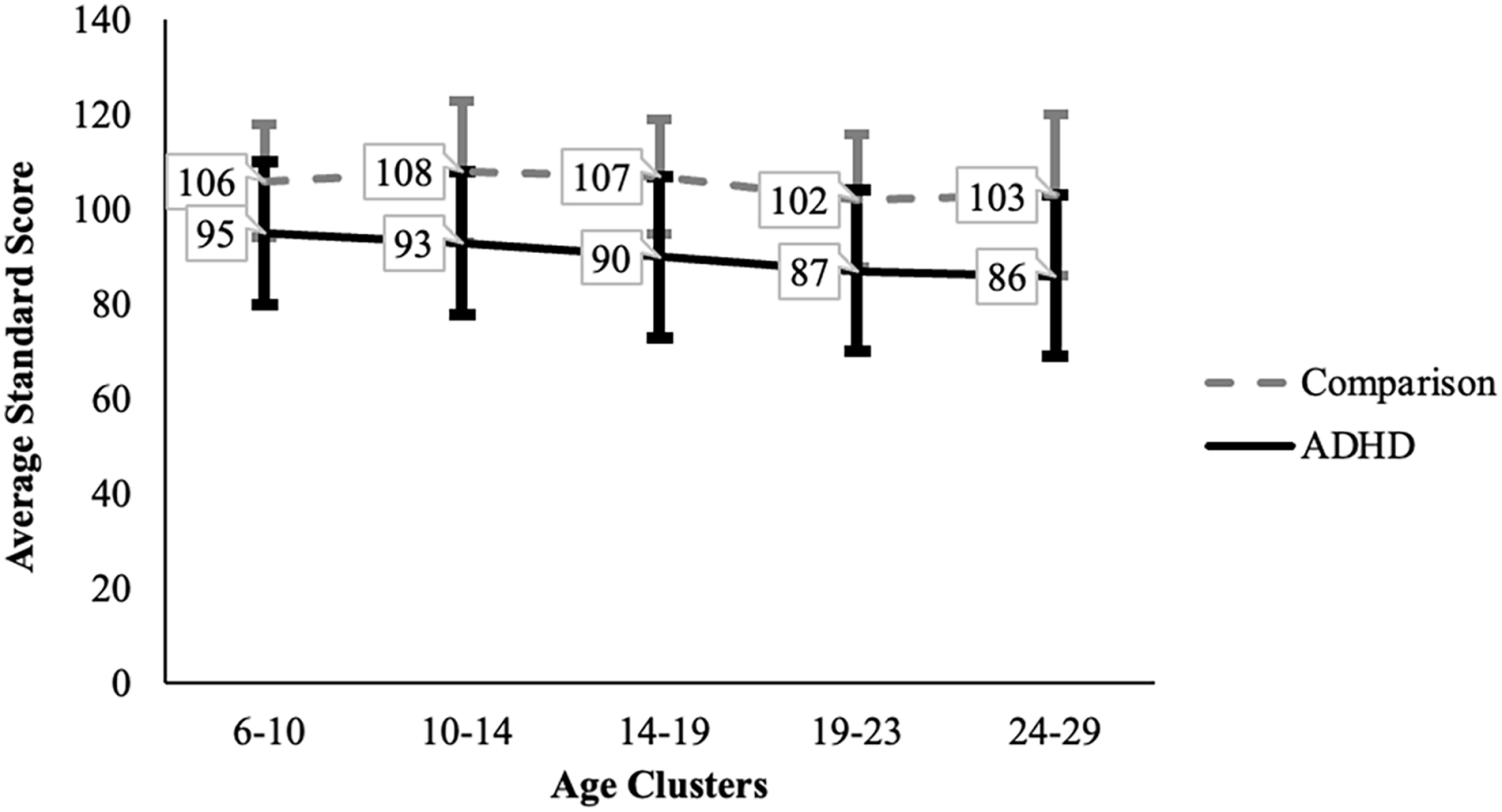
Average WIAT Math Achievement Standard Score by Age Cluster. *Note.* Error bars represent the standard deviations. ADHD = attention-deficit/hyperactivity disorder. Age clusters are ranges in years.

To illustrate change in math levels over time graphically, we calculated average standard scores at each age cluster (see Figure 1). Participants both with and without ADHD declined in average math scores over time, and the ADHD sample mean score was at the 18th percentile of national norms by adulthood (standard score = 86).

#### Childhood ADHD Diagnosis and Math Difficulty Proportions Over Time

Table 1 shows the percentage of girls meeting math difficulty criteria at each wave. At Wave 1, 1% to 15% of girls with ADHD exhibited childhood math difficulties, which increased to 22% to 48% at Wave 4. In the comparison sample, 0% to 7% of girls had childhood math difficulties at Wave 1, which increased to 4% to 15% at Wave 4. Also, of the girls with ADHD *without* math difficulties at Wave 1 who were retained through Wave 4, 22% to 39% revealed math difficulties by Wave 4 (39/99 girls with the 1 *SD* criterion; 37/110 with 1.5 *SD* criterion; 25/116 with 2 *SD* criterion). Of the comparison girls without math difficulties at Wave 1 retained through Wave 4, 4% to 14% revealed math difficulties by Wave 4 (1*SD*_N_ = 11/81, 1.5*SD*_N_ = 8/85, 2*SD*_N_ = 3/85). Next, of the girls with ADHD *with* math difficulties at Wave 1 who were retained through Wave 4, only 1 girl no longer had math difficulties at Wave 4 (1*SD*_N_ = 1/18, 1.5*SD*_N_ = 1/7, 2*SD*_N_ = 0/1). Of the four comparison girls with math difficulties at Wave 1 who were retained through Wave 4, two no longer had math difficulties at Wave 4 (1*SD*_N_ = 2/4, 1.5*SD*_N_ = 0/0, 2*SD*_N_ = 0/0).

**Table 1.** Percentage of Girls with Math Difficulties by Group and Wave.

	1 *SD* below the mean		1.5 *SD* below the mean		2 *SD* below the mean	
Wave	*ADHD*	*Comparison*	*ADHD*	*Comparison*	*ADHD*	*Comparison*
Wave 1	15% (21/137)	7% (6/88)	6% (8/137)	0% (0/88)	1% (1/137)	0% (0/88)
Wave 2	30% (37/123)	2% (2/81)	15% (19/123)	1% (1/88)	7% (9/123)	0% (0/81)
Wave 3	34% (44/128)	7% (6/84)	19% (24/128)	4% (3/84)	10% (13/128)	2% (2/84)
Wave 4	48% (57/120)	15% (13/85)	36% (43/120)	9% (8/85)	22% (26/120)	4% (3/85)

*Note*. ADHD = attention-deficit/hyperactivity disorder.

#### Growth in Math Achievement

Before using LGCM and FIML, we examined whether data met the missing at random assumption for FIML (i.e., someone having missing data in the variable being addressed by FIML is not associated with the value of the missing variable). First, we examined the amount of missing math data at each wave: Wave 1, 3 participants (1%); Wave 2, 24 participants (11%); Wave 3, 16 participants (7%); and Wave 4, 23 participants (10%). To verify this assumption, as in previous longitudinal SEM research from this data set (Porter et al., 2021), we leveraged data from proximal waves to estimate differences in the dependent variable between participants with and without missing data at a particular wave (e.g., to examine math scores in participants with Wave 2 missing math data, we used Wave 1 and Wave 3 math data). We found no significant differences in proximal math scores between participants with and without missing data at a given wave, suggesting data met the missing at random assumption. Missing data created by the developmental period restructuring approach were based on age and unrelated to math values, so by design met the missing at random assumption.

We next conducted unconditional growth models (i.e., examining the shape of the growth in math scores, *without* including any predictors) and considered four shapes of growth: linear, quadratic, cubic, and unspecified (via a latent basis curve model by which no shape of growth was imposed; see Table 2 for comparisons of these models). Table 2 shows that linear growth models fit better than quadratic and latent basis curve models. We set the loadings from the latent slope of the repeated math measures to 0, 1, 2, 3, and 4, representing the different points in time, and fixed the loadings from the latent intercept of the repeated measures to 1 to have a common reference point from which to measure growth. Table 3 shows the parameter estimates of this unconditional linear model, revealing significant intercept and slope variances, meaning that participants varied in both their initial math achievement scores and in their growth from childhood through early adulthood. The intercept-slope covariance was not significant, which indicates a lack of relation between initial math scores and rates of change in math scores (in other words, those with higher childhood math scores were *not* more likely to have more growth in math scores over time).

**Table 2:** Unconditional Latent Growth Curve Models of WIAT Math Achievement.

Variable	Linear growth model	Quadratic growth model	Cubic growth model	Latent basis curve model
χ^2^ *p*-value	.02	*Inadmissible solutions*		.01
RMSEA	.07			.08
CFI	.98			.98
TLI	.98			.97
SRMR	.07			.11

*Note*. For RMSEA and SRMR, lower values indicate better fit. For CFI and TLI, higher values indicate better fit. No fit information is available for the quadratic and cubic growth models as they resulted in inadmissible solutions. For information on inadmissible solutions, see Boomsma, 1985.

RMSEA = Root Mean Square Error of Approximation; CFI = Comparative Fit Index; TLI = Tucker-Lewis Index; SRMR = Standardized Root Mean Square Residual.

**Table 3.** LGCMs for Aim 1: Unconditional Growth Model and Model With Childhood ADHD Status Predicting Math Achievement.

	Unconditional growth model		Conditional growth model	
Variable	*B* (*SE B*)	β (*SE* β)	*B* (*SE B*)	β (*SE* β)
Means				
Intercept	99.220* (1.029)	7.345* (0.471)	30.483* (6.225)	2.303* (0.558)
Slope	−1.750* (0.268)	−0.757* (0.181)	−1.854 (2.438)	−0.906 (1.207)
Variances				
Intercept	182.460* (22.898)	1	45.202* (12.629)	0.258* (0.063)
Slope	5.338* (1.905)	1	4.046* (1.877)	0.967* (0.047)
Intercept-slope covariance	1.732 (4.837)	0.055 (0.162)	3.072 (3.982)	0.227 (0.358)
Intercept on				
ADHD			−4.646* (1.601)	−0.171* (0.060)
SES			1.021 (0.723)	0.077 (0.054)
FSIQ			0.687* (0.055)	0.752* (0.049)
Slope on				
ADHD			−0.688 (0.610)	−0.164 (0.145)
SES			0.039 (0.276)	0.019 (0.134)
FSIQ			0.004 (0.021)	0.030 (0.151)

*Note.* The unconditional growth model examines the parameters of the growth model of math scores *without* including any predictors, whereas the conditional growth model includes ADHD status and covariates as predictors of math intercept/slope. LGCMs = latent growth curve models; ADHD = attention-deficit/hyperactivity disorder; SES = socioeconomic status; FSIQ = Full-Scale Intelligence Quotient.

* *p* < .05.

#### Childhood ADHD Status and Growth in Math Achievement

Next, we conducted LGCM with ADHD as predictor of latent intercept and latent slope of math trajectories, covarying SES and full-scale IQ. Model fit was acceptable, χ^2^(16) = 33.04, *p* = .02; Root Mean Square Error of Approximation (RMSEA) = .06; Comparative Fit Index (CFI) = .98; Tucker–Lewis Index (TLI) = .98; Standardized Root Mean Square Residual (SRMR) = .06. Table 3 shows the parameter estimates of this conditional model (i.e., the model *with* predictors/covariates): Childhood ADHD was coded as 1 = *ADHD* and 0 = *comparison*, so the negative intercept coefficient of β = −0.17, *p* = .004 indicates that the expected math standard score at the first age cluster was lower for girls with ADHD than for comparisons. No variables significantly predicted the latent slope. Although participants with ADHD started with significantly lower math scores than comparisons, the nonsignificant slope coefficient β = −0.16, *p* = .26 shows that they did not show significantly slower math score growth than comparison girls. As in the unconditional model, the intercept-slope covariance was not significant (i.e., there was no significant relation between initial math scores and rates of change in math scores).

### Aim 2

#### Initial Regressions Between Predictors and Math at Wave 4

Regression results between Wave 1 predictors and Wave 4 math, covarying ADHD status and SES, and with Benjamini–Hochberg alpha corrections, revealed that global EF errors (with an additional covariate of PIQ, β = −0.14 *t* = −2.19, *p* = .03, ΔR^2^ Adj. = 0.02), PS (β = 0.34, *t* = 5.49, *p* < .001, ΔR^2^ Adj. = 0.10), and WM (β = 0.32, *t* = 4.78, *p* < .001, ΔR^2^ Adj. = 0.06) significantly predicted Wave 4 math. Thus, we included all three predictors in the LGCMs (see Supplement for exploratory analyses).

#### Predictors of Math LGCMs

Table 4 shows conditional growth curve model results with PS, WM, and global EF as predictors of the latent intercept and latent slope of math scores, covarying ADHD and SES (and PIQ in the global EF model). The PS model fit well, χ^2^(19) = 27.553, *p* = .092; RMSEA = .045; CFI = .987; TLI = .983; SRMR = .060. The other two models fit adequately: both WM: χ^2^(19) = 39.896, *p* = .003; RMSEA = .075; CFI = .964; TLI = .953; SRMR = .050; and global EF: χ^2^(18) = 35.595, *p* = .03; RMSEA = .053; CFI = .981; TLI = .974; SRMR = .049. Both PS (β = .367, *p* < .001) and WM (β = .303, *p* < .001) predicted initial math. Adjusting for ADHD and SES, which predicted the latent intercept in all models, a 1 *SD* increase in PS was associated with a .37 *SD* increase in initial (childhood) math. The same increase in WM was associated with a .30 *SD* increase in initial math. However, neither PS (β = .222, *p* = .090) nor WM (β = .105, *p* = .446) predicted rate of change in math.

**Table 4.** LGCMs With Childhood Predictors and Math Achievement (Aim 2).

	Processing speed (PS)		Working memory (WM)		Global executive functioning (Global EF)	
Variable	*B* (*SE B*)	β (*SE* β)	*B* (*SE B*)	β (*SE β*)	*B* (*SE B*)	β (*SE β*)
Means						
Intercept	72.905* (6.375)	5.408* (0.663)	96.037* (3.143)	7.308* (0.589)	48.056* (7.158)	3.615* (0.669)
Slope	−4.583* (1.955)	−2.125* (0.972)	−1.987* (0.960)	−0.889 (0.475)	0.936 (2.535)	0.445 (1.200)
Variances						
Intercept	106.816* (17.450)	0.588* (0.068)	111.457* (18.028)	0.645* (0.070)	65.737* (15.621)	0.372* (0.071)
Slope	4.315* (1.894)	0.928* (0.066)	4.840* (1.916)	0.969* (0.043)	3.845 (2.045)	0.868* (0.100)
Intercept-slope covariance	−0.790 (4.576)	−0.037 (0.206)	0.735 (4.617)	0.032 (0.204)	2.662 (4.574)	0.167 (0.336)
Intercept on						
ADHD	−10.374* (1.856)	−0.376* (0.065)	−10.761* (1.944)	−0.406* (0.070)	−7.741* (1.783)	−0.284* (0.065)
SES	2.399* (0.886)	0.177* (0.065)	2.297* (0.946)	0.171* (0.070)	2.054* (0.803)	0.154* (0.060)
PIQ					0.544* (0.061)	0.602* (0.062)
PS	0.316* (0.058)	0.367* (0.064)				
WM			2.203* (0.554)	0.303* (0.073)		
Global EF					0.202 (4.641)	**0.003** (0.065)
Slope on						
ADHD	−0.464 (0.563)	−0.105 (0.126)	−0.526 (0.583)	−0.117 (0.128)	−0.458 (0.614)	−0.106 (0.142)
SES	−0.001 (0.271)	0.000 (0.125)	0.052 (0.285)	0.023 (0.125)	0.012 (0.279)	0.005 (0.132)
PIQ					−0.014 (0.021)	−0.098 (0.151)
PS	0.031 (0.018)	**0.222** (0.131)				
WM			0.130 (0.168)	**0.105** (0.138)		
Global EF					−3.836* (1.629)	−0.342* (0.155)

*Note.* Focal parameters are in bold. LGCMs = latent growth curve models; ADHD = attention-deficit/hyperactivity disorder; SES = socioeconomic status; PIQ = Performance Intelligence Quotient from the Wechsler Intelligence Scale for Children-III.

* *p* < .05.

Global EF was not significantly related to initial math when adjusting for covariates (β = 0.003, *p* = .965), but it predicted growth in math achievement over time (β = −0.342, *p* = .027). Because global EF was coded such that higher scores represent more errors, the negative coefficient indicates that poorer global EF predicts less increase in math over time. The visuo-spatial covariate, PIQ, predicted initial math but did not change over time in math (see Table 4).

### Secondary Tests

For secondary tests, including Aim 1 without IQ as a covariate, Aim 2 exploratory regression models (see Method), Aim 2 models with IQ as a covariate, models with education level as a covariate, and exploration of correlations between Aim 2 predictors, see Supplement Materials.

## Discussion

We sought to characterize 16-year math developmental trajectories in girls with and without childhood ADHD and identify childhood cognitive predictors of trajectories. Girls with ADHD started in childhood and ended in adulthood with significantly lower math scores than the comparison sample (see Gordon & Hinshaw, 2020 for cross-sectional comparison in adulthood). Both girls with and without ADHD declined in average standard scores from childhood to adulthood, and contrary to our hypothesis, the group difference in rates of change was not statistically significant. Change in math over time was also not related to initial math skill levels.

Although the change in math scores over time in the comparison sample was clinically nominal, the low math scores of the ADHD sample in childhood, plus their decline across development, yielded average scores by adulthood in the 18th percentile of national norms, compared to the 37th percentile in childhood. We consider this finding clinically meaningful for several reasons. First, considering the proportion of math difficulties in the ADHD sample (and depending on the learning difficulty criteria used), math difficulty rates increased from 1% to 15% in childhood to 22% to 48% in adulthood. There was a lesser increase in the comparison sample (from 0% to 7% in childhood to 4% to 15% in adulthood). Second, from another angle, a notable portion of girls with ADHD *without* math difficulties in childhood revealed difficulties later in development (i.e., of the girls without childhood math difficulties, 22% to 39% of girls with ADHD gained math difficulties by adulthood, compared to 4% to 14% of comparison girls). Moreover, childhood math difficulties were persistent across 16 years. When considering the 22 girls with childhood math difficulties in the full sample, only 3 girls no longer exhibited math difficulties in adulthood.

The math difficulties experienced by these women may well affect their daily life functioning. Indeed, the WIAT math problem-solving test involves daily life applications such as money, time, measurement, and interpreting graphs and charts. Some evidence exists linking math test scores to daily life financial functioning and other daily life skills linked to math (e.g., measurement, interpretation of graphs, Agarwal & Mazumder, 2013; Johnson & Blalock, 1987). Moreover, academic skills are linked to several domains of later well-being in girls with ADHD (e.g., Guendelman et al., 2016; Owens & Hinshaw, 2016, 2020).

Why did both groups decline in math standard scores over time? Considering our comparison sample, given that their average math score started a few points above the population average at Wave 1 and became closer to average across time, their decline might simply reflect regression to the mean. Alternatively, societal barriers to advancement in mathematics faced by women might have reduced opportunities for the women in our study to have practiced math, resulting in standard score declines (i.e., slower growth) relative to the population (mix-gendered) average in both the ADHD and comparison groups.

It is surprising that contrary to our expectations, ADHD did not predict math slope, particularly given that global EF predicted math slope and given that EF difficulties are commonly associated with ADHD. We note that not all children with ADHD exhibit EF deficits, and indeed, there is heterogeneity in EF presentations in children with ADHD (e.g., Willcutt et al., 2005). Thus, it could well be that because our ADHD predictor was categorical, and because our EF predictor was continuous, those with higher EF in our ADHD sample may have diluted the relation between ADHD and math growth. In contrast, girls with very low EF might have been highlighted in the model with the continuous measure of EF. Considering another possible explanation, because ADHD is associated with lower math scores at all time points including baseline, features associated with ADHD (beyond just EF) may contribute to math deficits consistently over time, whereas the specific relation between global EF and math might strengthen across development. This supposition is consistent with our finding that global EF predicted math slope and adult math scores but *not* initial childhood math scores (see below for more detail on the relation between EF and math growth over time).

Addressing our second aim: Results of preliminary regressions revealed that the childhood cognitive measures we examined (global EF, WM, PS) were all significant predictors of adult math. These predictive relations were not different between the ADHD and comparison groups. In our growth curve models, although we expected all cognitive measures to predict change in math scores over time, only the measure of childhood global EF significantly predicted math slope (note that inclusion of several covariates enhanced the stringency of our analyses). The global EF measure highlights the higher-order EF skill of planning/strategizing (Sami et al., 2004) and may recruit relevant lower-order cognitive abilities that work together to enable planning to occur, such as attention, inhibitory control, visuo-spatial skills, WM, and PS. Indeed, all of these skills may well be useful for successful math performance across development.

Yet WM, PS, and visuo-spatial skills (i.e., PIQ) did not predict math growth. As well, covariation of visuo-spatial skills did not alter the global EF findings. Moreover, previous research suggests that measures of response inhibition and sustained attention do not predict long-term math (Miller et al., 2012). We believe these data suggest that a key higher-order EF—planning—is a good candidate for future research on long-term math growth. In one promising direction, there is evidence of malleability of the planning skills of adolescents with ADHD from clinical intervention such as the Homework, Organization, and Planning Skills (HOPS) program (Breaux et al., 2019; Langberg et al., 2012) and the Challenging Horizons Program (CHP) (Evans et al., 2016), which have been shown to improve organizational and academic functioning. Still, more research is needed on applications to math. Of note, the relation between EF and later math was not moderated by diagnostic group, indicating that childhood EF might be a transdiagnostic indicator of later long-term math growth.

The EF impairments in girls with ADHD tend to persist through adolescence and adulthood even when ADHD symptoms have remitted (Gordon & Hinshaw, 2020). Thus, it could well be that as math complexity increases across development, global EF increases in importance for math performance. As well, as girls mature into adulthood, their parents and teachers might take less responsibility for helping them plan how to approach schoolwork/homework, thereby increasing recruitment of girls’ own EF skills to facilitate classroom success. Delineating specific dynamics of how global EF might play a role *during* the process of math problem-solving and math learning in individuals with ADHD is a key area for future research.

Knowledge of these long-term math trajectories may help families/schools plan for the future. Providing academic supports may be important for girls with ADHD/EF difficulties *before* adolescence/adulthood, as math problems may emerge or persist. This approach differs from standard practices for learning interventions, which reflect current rather than anticipated future performance (e.g., Greenwood et al., 2011). Early detection of learning difficulties is crucial, given greater success of earlier rather than later intervention (Fletcher et al., 2018). For early support of math skills specifically, recent recommendations support early discernment of the types of math skills difficulties in each child, followed by intensive instruction tailored to those specific math difficulties. Fuchs et al. (2021) provide more information about this approach and present a helpful list of relevant intervention steps and instructional design tips.

Why did PS and WM fail to predict the development of math over time? Given that PS and WM predicted both initial and adult math but not change in such scores, it may be that they are more important for math performance during testing than for math *learning* over time. It could also be that they are similarly important for math across different developmental periods. Regardless, given that their relations with math, monitoring WM and PS in girls with ADHD and providing relevant accommodations (e.g., breaking down instructions into manageable pieces) for girls with WM and PS difficulties could enhance successful math performance.

Limitations of our measures might also help explain results. Considering PS, we used a measure that is nonlinguistic and relatively simple. Yet it may be that more complex measures of PS that recruit language are more strongly linked to math difficulties (e.g., Moll et al., 2016). Still, we note that our relatively basic PS measure predicted math skills 16 years later. As for math measurement, we used two different versions of the WIAT at different measurement occasions. Despite good concordance between versions and despite our adjusting for score differences, slight differences in test content or standardization may have been a possible confound. Finally, our sample is not necessarily representative nationally or internationally.

Future research on math trajectories should also include boys. It would also benefit from examination of parenting, stereotype threat, math anxiety, psychiatric comorbidities, and peer/familial/teacher beliefs. Clinical trials are needed to examine the influence of stimulant medication and psychoeducational interventions. Investigation of other academic trajectories, including reading skills, is also critical. Although math problem-solving has the benefit of reflecting some “real-world” math applications, future research should also include other math measures, such as the Numerical Operations subtest of the WIAT (which measures math calculation skills). Measurement of “daily life” EF (particularly EFs as they are used during math problem-solving or learning math) in relation to math trajectories is also a priority. In addition, above and beyond neuropsychological measures of EF, future research should examine school impairment (e.g., special education involvement, grade retention, grade point average, suspensions) in relation to math trajectories, because school impairment importantly captures the daily life burdens associated with ADHD (Barkley, 2016).

More globally, although girls perform well in math in school when equal opportunities are available (e.g., Else-Quest et al., 2010), evidence also suggests that societal gender parity in school/career/mentorship opportunities, as well as stereotypes and societal perceptions, influence girls’ math performance and pursuit of continued higher math education (e.g., Else-Quest et al., 2010; Good et al., 2008). As well, attitudes about ADHD may also influence long-term math performance (e.g., Foy, 2018). We raise these points to emphasize that the low math scores, on average, exhibited by our ADHD sample through early adulthood should not give rise to further stereotypes or low expectations. Instead, early detection/intervention should be prioritized.

## Supplemental Material

sj-docx-1-ldx-10.1177_00222194241301044 – Supplemental material for Mathematics Achievement in Women With and Without ADHD: Childhood Predictors and Developmental Trajectories Into Adulthood Supplemental material, sj-docx-1-ldx-10.1177_00222194241301044 for Mathematics Achievement in Women With and Without ADHD: Childhood Predictors and Developmental Trajectories Into Adulthood by Laura N. Henry, Rachel A. Gross and Stephen P. Hinshaw in Journal of Learning Disabilities

sj-docx-2-ldx-10.1177_00222194241301044 – Supplemental material for Mathematics Achievement in Women With and Without ADHD: Childhood Predictors and Developmental Trajectories Into Adulthood Supplemental material, sj-docx-2-ldx-10.1177_00222194241301044 for Mathematics Achievement in Women With and Without ADHD: Childhood Predictors and Developmental Trajectories Into Adulthood by Laura N. Henry, Rachel A. Gross and Stephen P. Hinshaw in Journal of Learning Disabilities

sj-docx-3-ldx-10.1177_00222194241301044 – Supplemental material for Mathematics Achievement in Women With and Without ADHD: Childhood Predictors and Developmental Trajectories Into Adulthood Supplemental material, sj-docx-3-ldx-10.1177_00222194241301044 for Mathematics Achievement in Women With and Without ADHD: Childhood Predictors and Developmental Trajectories Into Adulthood by Laura N. Henry, Rachel A. Gross and Stephen P. Hinshaw in Journal of Learning Disabilities
