# Supplementary material for: Mathematics Achievement in Women With and Without ADHD: Childhood Predictors and Developmental Trajectories Into Adulthood
Source: J Learn Disabil. 2025 Jan 6;58(6):431–44. doi: 10.1177/00222194241301044 (PMC12228839; doi:10.1177/00222194241301044)
Supplement: sj-docx-1-ldx-10.1177_00222194241301044 – Supplemental material for Mathematics Achievement in Women With and Without ADHD: Childhood Predictors and Developmental Trajectories Into Adulthood [file sj-docx-1-ldx-10.1177_00222194241301044.docx]

**Supplemental Results: Secondary Tests**

***Aim 1 without covarying FSIQ.*** We examined childhood ADHD and SES as predictors of math intercept/slope. Focal results did not change: ADHD predicted intercept but not slope.

***Exploratory Regressions.*** First, ADHD status was not a significant moderator of any relations between childhood cognitive predictors and adult math. Regarding our second exploratory question, when adding Wave 1 reading as an additional covariate, relations between cognitive predictors and Wave 4 math remained significant, with some small reductions in beta coefficients and effect sizes: global EF EPS (β= -0.14, t=-2.31, *p* =0.022, ΔR^2^ Adj. =0.02), PS (β=0.27, t=4.49, *p* < 0.001, ΔR^2^ Adj. = 0.06), WM (β=0.26, t=4.13, *p* < 0.001, ΔR^2^ Adj. =0.04).

***Supplemental IQ Covariate Models.*** See Table S1. Our focal results did not substantially change: WM and PS predicted math intercept, and global EF predicted math slope but not intercept. Although WM again did not predict slope, PS significantly predicted slope. It may be that models without childhood ADHD status as a covariate are less stringent.

***Education Level Covariate Model.*** Given that global EF predicted math slope, we added education level as an additional covariate to the previous three covariates. Core findings remained the same: Global EF predicted math slope but not math intercept.

***Covariation Between Predictors.*** Global EF and PS were significantly correlated, *r*(217) = -0.15, *p*=0.021, as were global EF and WM, *r*(189)=-0.43, *p*<.001. WM and PS were not significantly correlated, *r*(195) = 0.10, *p*=0.158. First, we reconstructed our primary regression model with EF and its covariates to also include PS. Both EF and PS remained significant predictors of Wave 4 math. Next, we reconstructed the same primary EF model with the addition of WM. Again, both WM and EF remained significant predictors of Wave 4 math, suggesting independent contributions of each cognitive predictor to adult math achievement.
