# Supplementary material for: Mathematics Achievement in Women With and Without ADHD: Childhood Predictors and Developmental Trajectories Into Adulthood
Source: J Learn Disabil. 2025 Jan 6;58(6):431–44. doi: 10.1177/00222194241301044 (PMC12228839; doi:10.1177/00222194241301044)
Supplement: sj-docx-2-ldx-10.1177_00222194241301044 – Supplemental material for Mathematics Achievement in Women With and Without ADHD: Childhood Predictors and Developmental Trajectories Into Adulthood [file sj-docx-2-ldx-10.1177_00222194241301044.docx]

|  | **ADHD** | | **Comparison** | |
| --- | --- | --- | --- | --- |
|  | *Mean or %* | *SD* | *Mean or %* | *SD* |
| **Demographic** |  |  |  |  |
| Age (months) | 115.6 | 20.2 | 113.2 | 19.8 |
| Total annual family income | 6.2 | 2.7 | 6.7 | 2.5 |
| Maternal education level* | 4.7 | 1.0 | 4.9 | 1.0 |
| Caucasian (%) | 56.4 |  | 46.6 |  |
| Black (%) | 27.9 |  | 26.1 |  |
| Latina (%) | 10.7 |  | 11.4 |  |
| Asian-American (%)** | 4.3 |  | 15.9 |  |
| Native American (%) | 0.7 |  | 0 |  |
| Public assistance (%) | 15.0 |  | 11.4 |  |
| Two parent household (%) | 65.7 |  | 77.3 |  |
| **Background (%)** |  |  |  |  |
| Low birthweight (<2,500 g) | 10.5 |  | 10.7 |  |
| History of grade retention** | 17.3 |  | 3.4 |  |
| Speech/language problems *** | 27.0 |  | 7.0 |  |
| Adopted*** | 21.4 |  | 4.5 |  |
| **Comorbidities (%)** |  |  |  |  |
| ODD*** | 61.9 |  | 6.8 |  |
| Anxiety disorder*** | 27.0 |  | 3.4 |  |
| Depression/dysthymia* | 8.2 |  | 0.0 |  |
| **Study variables** |  |  |  |  |
| Full scale IQ*** | 99.7 | 13.6 | 112.0 | 12.7 |
| Processing speed*** | 99.9 | 15.8 | 108.1 | 14.3 |
| Working memory** | 4.3 | 1.7 | 5.0 | 1.9 |
| Global EF EPS*** | 0.35 | 0.19 | 0.22 | 0.15 |
| Math Reasoning*** | 97.5 | 14.3 | 109.1 | 13.8 |
| Word Reading*** | 101.8 | 14.4 | 112.0 | 12.6 |

*Note:* ODD = oppositional defiant disorder. For comorbidities, the parent DISC–IV (Shaffer et al., 2000) was used to assess “current” categorical child psychiatric diagnoses. For the DISC–IV, “current” means any time between 12 months before the interview until the date of the interview. For anxiety disorders, one or more of the following was counted: social phobia, agoraphobia, panic disorder, separation anxiety disorder, generalized anxiety disorder, post traumatic stress disorder, or obsessive compulsive disorder. Specific phobias were not counted. To examine group differences, chi-square tests were used for categorical variables and t-tests for continuous variables.

*p<.05

**p<.01

***p<.001
