# Supplementary material for: Mathematics Achievement in Women With and Without ADHD: Childhood Predictors and Developmental Trajectories Into Adulthood
Source: J Learn Disabil. 2025 Jan 6;58(6):431–44. doi: 10.1177/00222194241301044 (PMC12228839; doi:10.1177/00222194241301044)
Supplement: sj-docx-3-ldx-10.1177_00222194241301044 – Supplemental material for Mathematics Achievement in Women With and Without ADHD: Childhood Predictors and Developmental Trajectories Into Adulthood [file sj-docx-3-ldx-10.1177_00222194241301044.docx]

|  | Processing Speed | Working Memory | Executive Function/Planning |
| --- | --- | --- | --- |
| Means  Intercept  Slope | 0.935 (0.546)  -1.873 (1.164) | 1.835* (0.582)  -1.394 (1.075) | 1.708* (0.546)  0.009 (1.194) |
| Variances  Intercept  Slope | 0.365* (0.066)  0.930* (0.068) | 0.388* (0.070)  0.971* (0.046) | 0.245* (0.068)  0.869* (0.105) |
| Intercept-slope covariance | 0.175 (0.292) | 0.175 (0.281) | 0.305 (0.462) |
| Intercept on  IQ  SES  PS  WM  Global EF | 0.656* (0.056)  0.081 (0.058)  0.219* (0.061) | 0.685* (0.057)  0.015 (0.132)  0.194* (0.068) | 0.823* (0.049)  0.085 (0.056)  -0.051 (0.057) |
| Slope on  IQ  SES  PS  WM  Global EF | -0.108 (0.144)  0.021 (0.130)  0.287* (0.141) | 0.023 (0.139)  0.015 (0.132)  0.160 (0.145) | -0.053 (0.152)  0.010 (0.138)  -0.375* (0.156) |

**p* < .05.

*Note.* Standardized model results are shown, with standard errors in parenthesis, β *(SE* β*).* For the model with global EF as a predictor, Full scale IQ was used as a covariate. Because PS and WM were both measured by the IQ test (WISC-III), we covaried the sum of verbal comprehension index (VCI, including subtests: Information, Similarities, Arithmetic, and Comprehension) and the Perceptual Organization Index (POI, including subtests: Picture Completion, Picture Arrangement, Block Design, Object Assembly) in these models rather than full scale IQ, given that VCI and POI do not overlap with PS and WM.
